# Reflecting on twenty years of international agreements concerning water governance: insights and key learning

**DOI:** 10.1007/s10784-022-09564-9

**Published:** 2022-02-19

**Authors:** Naho Mirumachi, Margot Hurlbert

**Affiliations:** 1 Department of Geography, King’s College London, 40 Aldwych, London, WC2B 4BG UK; 2 Centre for the Study of Science and Innovation Policy, Johnson-Shoyama Graduate School of Public Policy, University of Regina, 332.4- 2155 College Avenue, Regina, SK S4P 4V5 Canada

**Keywords:** Equity, Power, Transboundary waters, Water governance, Water, Sanitation and hygiene (WASH)

## Abstract

The purpose of this article is to examine the research advanced in the journal, International Environmental Agreements: Politics, Law and Economics that represents key insights into international agreements on water and their political, legal, economic and cross-disciplinary dimensions for water governance. The article analyses evidence and lessons learnt over the last twenty years to inform policy through a review of theoretical advances, innovations in principles and policy instruments, outcomes of problem-solving and knowledge gained regarding water agreements and associated institutions. Important international agreement principles of no significant harm and economic frames of water as a ‘commons’ advance equity and community of interest in relation to water. The studies on water, sanitation and hygiene point to the ways the role of the state can be advanced in achieving Sustainable Development Goals and in complex contexts of water scarcity and public private partnerships. Cross-disciplinary learnings substantiate the existence and utility of multiple water frames in legal arrangements and use of multiple policy instruments. Cross-disciplinary insights are significant in addressing equity, whether through the nascent development of water indicators or in advancing social learning. Water governance frameworks increasingly focus on adaptation by incorporating multiple stakeholders. These findings that advance equity and inclusivity are tempered by crucial lessons in our understanding of the very contested, power-laden nature of water governance that impact agency at multiple scales and policy coordination across sectors of water, food and energy.

## Introduction

Since *International Environmental Agreements: Politics, Law and Economics* (INEA) was launched in 2001, there have been major global developments dealing with water issues. The journal’s creation coincided with the Millennium Development Goals release, which included ambitions to “Halve, by 2015, the proportion of the population without sustainable access to safe drinking water and basic sanitation”. The twentieth year of the journal comes at a time when the United Nations General Assembly is preparing to mark a milestone on five decades of progress since the 1972 United Nations Conference on the Human Environment, more widely known as the Stockholm Conference. There is sustained global attention to address issues of water access and allocation, focusing both on quantity and quality, as well as human and ecosystem health. Water is arguably at the top of the agenda as none of the Sustainable Development Goals (SDGs) can be met without access to adequate and safe water [and the world is off track on meeting this goal (Sadoff et al., 2020)]; the water crisis has been ranked as one of the top five risks since 2015 by the World Economic Forum (WEF, 2019). In relation, the scholarship on water issues has expanded in depth, breadth, and quantity, and important trends have been captured in the INEA journal.

The purpose of this article is to examine the research advanced in INEA that represents key insights into international agreements on water and their political, legal, economic and cross-disciplinary dimensions for water governance. The article synthesises and analyses evidence to inform policy through a review of theoretical advances, innovations in principles and policy instruments, outcomes of problem-solving and lessons learnt regarding these agreements and associated institutions concerning water governance. The review of INEA scholarship highlights lessons learnt from articles under the scope of the journal, rather than provide a comprehensive synthesis of advances and innovations in the broad field of water governance, of which there are many elsewhere. By doing so, this article contributes to this special issue, which consolidates advances made in INEA with a focus on the effectiveness, efficiency and fairness of environmental policies (Gupta et al., this issue).

The analysis of this article draws on 58 articles published between 2001 and 2020. The collective insights of the studies show that the INEA water scholarship has brought to the fore the political nature of managing and governing water. Put differently, international water agreements themselves are products of contestations and struggles on modes of water governance. It is argued that there is further scope for INEA water scholarship to advance and generate novel insights on alternative discourses and water governance arrangements. In particular, cross-disciplinary insights that bridge legal, economic and political perspectives provide sharper thinking and perspectives on ways to address equity.

In the following sections, the article first presents a brief methodology of the review. This section is followed by a substantial analysis of lessons learnt organised around (i) the nature of international environmental agreements; (ii) legal dimensions; (ii) economic insights; (iii) politics and power; (iv) and cross-disciplinary insights. Finally the article concludes with implications of lessons learnt for new opportunities for research directions.

## Method and criteria for analysis

Since its first issue in 2001 until 2020, there have been 58 articles published on the theme of water in INEA. These articles were first selected after a search for the word ‘water’ in keywords and abstracts. The articles were then categorised thematically using their keywords into the following: water, law, economics, justice, politics, agency, science-policy, climate change, market mechanisms and trade investment. Articles included at least two combinations of keywords, with some including three for categorisation. We studied these 58 articles in depth to determine lessons learnt and the new knowledge generated in each article. Of these, 39 articles examine transboundary water contexts, reflecting the journal’s key concern on international agreements on the environment. From our review, studies concerned problems or policy challenges relating to the categorisations of the journal: international agreements, law, economics and politics. In some cases, these problem types were not exclusive, and the focus of the articles touched upon multiple problem dimensions. The water scholarship of INEA tends to probe four lines of inquiry, formulating research questions around (1) international environmental agreement governance frameworks; (2) law and discourse of water governance; (3) neoliberalisation of water and role of markets; and (4) politics and agency. We expanded our review by analysing 15 additional articles closely associated with the INEA articles because they had been directly cited in the INEA articles or were highly related to the topic and in some instances more recently published, in order to provide further context. We did not consider articles outside of INEA as the purpose of this article is to synthesise the INEA studies and key findings over the past two decades, serving the aims of this special issue.

## International environmental agreements of water: lessons learnt

### Insights on international environmental agreements

In INEA scholarship, many studies clarify the principles that drive better processes and outcomes in international environmental agreements, particularly in transboundary river basin contexts. For example, there is work that speaks to wider debates on treaty design, which has been discussed extensively within hydropolitics research (e.g. Dinar et al., 2019; Mitchell et al., 2015). There are articles that reviewed how the scope and function of transboundary water treaties have changed over time globally (Giordano et al., 2014; Zawahri et al., 2016) or regionally (Jacobs, 2012; Kistin et al., 2009). Other case studies traced cooperative political efforts in the Iberian region (Lopes, 2012) or between China and India (Xie & Jia, 2017). Several studies also examined enabling factors of effectiveness (Dieperink, 2011; Jafroudi, 2018; Johns et al., 2018). Governance frameworks remain a key concern at the national level, with analyses highlighting the experiences in reforming institutions and managing practices in both low-income and high-income economies, such as Zimbabwe and South Africa (Jaspers, 2001) and Norway (Rosendal et al., 2019).

First, a key lesson learnt in the last twenty years highlights that international environmental agreements and management practices (at any spatial scale) will not necessarily hold in the future. For example, the design of treaties needs to be climate resilient with political mechanisms built in for flexibility in negotiating water allocation (Jafroudi, 2018). While links between groundwater and climate change are increasingly understood, international institutions on groundwater and on climate change have developed independently. Furthermore, these institutions have not developed a substantive link between each other (Gupta & Conti, 2017). As a result, there are contradictions and differences between international agreements, the framing of the climate and water problem, and their key norms and processes. The theme of dealing with uncertainty is echoed within the wider literature, suggesting that policy be made more flexible. For example, it is suggested that international agreements and treaties develop both water allocation and water quality measures which are adaptable and include scope for dealing with extreme events (Cooley & Gleick, 2011). At the national level, the Australian experience indicates that policy design and implementation need to ensure there are options to allow for adjustments and to reduce costly measures that lock-in future water allocation: “current political fascination with technical efficiency innovations to grow total water supply” is unrealistic and blind to changing social expectations (Loch et al., 2020: 12).

Second, if existing agreements are ill equipped for future contexts, then issues of equity arise. A lesson learnt is that international agreements need to better grapple with identifying and addressing equity. Communities and ecosystems can become vulnerable when agreements, underpinned by international law, might appear as cooperative decision-making, but effectively allow for state-centric development projects (Fox & Sneddon, 2007). This shows that relying on agreements alone may not yield results without causing irreversible harm, especially to ecosystems (Moynihan & Magsig, 2020). As will be further analysed below, INEA studies have adopted political lenses to understand the mechanisms of inequality. The emphasis on equity is echoed in wider scholarship as a “a first and fundamental step” of water governance (Hoogesteger & Wester, 2015: 118). There are empirical studies like that of Western India (Argade & Narayan, 2019) where equity is instrumentalised into a checklist, making participatory governance largely meaningless. In Tanzania, while a legal system of water permits has been established, the level of knowledge, technology and finance disposable to water users means disparity in equity of access, which is further compounded by issues of lack of monitoring compliance (Komakech & Bont, 2018). In the Middle East and North Africa region, experiences have shown that seeking government efficiency or accountability has not led to addressing equity, despite it seeming like a sensible approach. It is argued that policy instruments can only succeed if power dynamics consisting of influential actors and vested interests are taken into account (Closas & Villholth, 2020).

### Insights on law

There are three discrete lessons learnt regarding (a) the role of principles in treaty design; (b) the tension between the state and private sector in relation to water, sanitation and hygiene (WASH); and (c) the role of regulatory instruments in treaty design. First, the INEA literature concludes that the adoption of multiple principles surrounding water allocation and access aids consideration of equity. Principles of fairness and transparency are buttressed through the role of data and information exchange (Gerlak et al., 2011; Kotogaianni et al., 2006). It has been argued that key principles such as the no harm principle is being complementary with other principles, suggesting that a ‘community interest’ approach to transboundary waters avoids alleged priority of interests and subservience to other principles such as equitable and reasonable utilisation and cooperation (Tanzi, 2020). Clearly, the interpretation and application of the no significant harm principle has expanded over time, requiring not only legal but also political and economic considerations to place it into specific contexts (McIntyre, 2020). There are multiple temporal and spatial implications that can be included in a refined understanding of harm (Gupta & Schmeier, 2020). Thus, it is necessary for improved application of the principle in a particular context with clearer definitions of transboundary harm (Ziganshina & Janusz-Pawletta, 2020). This principle is now considered as supplementing the human right to water, such that states and individuals are better protected (Spijkers, 2020). There is synergistic practice of prior notification and the no harm principle, which allows for a structured exchange over time and the opening of a window of opportunity for cooperation and water diplomacy (Schmeier, 2020). The way in which principles support equity is important because of the structural factors constraining allocation or access to states and people.

The INEA literature documents legal discourses that impede equity, the no significant harm principle, and community of interest. The control of water resources, or hydro-hegemonic control, is in part enabled by discursive constructs of water scarcity and the subsequent justifications to address this problem (Zeitoun & Mirumachi, 2008; Zeitoun et al., 2011). The discourses are effective in curtailing attempts at challenging existing legal water priority agreements. The politics of river basin development is influenced by discourses around infrastructure (Warner, 2012), contested territory (Conker & Hussein, 2020), and the role of water for peace and stability (Nagheeby & Warner, 2018). Consequently, it has been argued that the use of these narratives need to be revised and re-examined with a view of equity, particularly from the perspective of those less powerful (Zeitoun et al., 2011).

In contexts of WASH of peoples, the principle of human rights to water and sanitation has evolved as a major dimension of the global framework of water governance. Despite the relatively small number of studies directly focusing on WASH (four out of 58 articles), the INEA literature provides critical points of reflection on the diffusion of the principle as well as the mechanisms through which equity is ensured. There has been a global expansion of the recognition of rights, in which judicial and quasi-judicial bodies have been instrumental. Nevertheless, courts are subject to political, social and economic contexts which give meaning and content to the rights (Obani, 2015). This global expansion also includes new legal frameworks such as the EU Water Framework Directive that underpins drinking water protection across a large number of heterogenous states (Jiménez et al., 2018). There is now progressive understanding of the intersection of international water law and international human rights law so that states and individuals are better served (Spijkers, 2020). The principle of human rights to water and sanitation is further made significant as its development is an increasingly important site of climate change adaptation law (Hurlbert, 2020). Considering that equality and non-discrimination are foundational to these human rights, the incorporation of such principles enhances debates on equity. From a national and international policy perspective, it is evident that these human rights offer important foundations that provide toeholds to improving access to safe drinking water and safely managed sanitation services, which underpin SDGs.

An important lesson learnt is that regulatory instruments are taken up by an increasing variety of actors but this also causes gaps and challenges of treaty implementation. Although policies and guidance exist, there is a gap in legally binding instruments as environmental impact laws and human rights laws do not go far enough. Regulatory instruments are in abundance, ranging from river basin organisations (Schmeier et al., 2016), valuation (Macatangay & Rieu-Clarke, 2018), environmental monitoring (Giordano et al., 2016) and legal tools (Holmatov et al., 2016). Advances in INEA scholarship has provided sharper attention to the legal solutions utilised by not only the state but also non-state actors. The heterogeneity of actors and the varied interactions continually adapt and evolve transboundary water systems. Consequently, rivers are complex adaptive systems in which policy needs are shaped through cultural, ethical and other normative features (Meissner & Jacobs, 2016). This insight suggests that regulatory instruments do not operate in a vacuum. When it comes to dealing with situations involving non-state actors, the role and legal obligations of private companies involved in hydropower projects are still underdeveloped, despite the promise offered by the principle of no significant harm in international law (Rieu Clarke 2020). Even if such principle is taken up, there are many challenges persisting in its interpretation, elaboration and operationalisation, and ultimately implementation (Schmeier & Gupta, 2020). This point about the effectiveness of regulatory instruments amid stakeholder variation is pertinent especially in a context of climate change. Considerations of multiple actors and how they contribute to implementing legal principles are relevant when large-scale infrastructure such as irrigation and dam projects in transboundary basins are often built by private enterprises of public private partnerships (PPPs).

Furthermore, disputes between investors and states highlight this challenge of treaty and principle implementation. Obani’s (2015) review of 65 WASH cases illustrates the disconnect often experienced between human rights that are obligations of the state, and international law concerning corporate activity. Obani documented that the court imposed obligation of the state to provide WASH in the context of service disruption. This finding has been more recently confirmed by Lark (2021) in a study of investments in WASH by the private sector, which has been regarded as particularly contentious. For example, in the case of Argentina, the government adopted measures to ensure access to WASH during the 1998–2002 economic crisis. Nevertheless, investors in WASH services disputed this as an interference with their investment. Since 1997, there have been 62 investor versus state dispute settlements. Investor-state dispute settlement mechanisms reveal how investors are able to pursue their rights in what are often periods of crisis. While in several cases investor’s rights predominated, in others the tribunals recognised states must advance both WASH and investor’s rights (Lark, 2021). A lesson to be drawn is that although WASH rights in emerging court cases can exist without expressed forms of recognition in national laws, it is advisable so that legislation in national laws can address limitations of fragmented legal sources of WASH rights.

### Insights on economics

Insights from INEA scholarship suggest that refining economic tools is useful but other complementary approaches are needed to realise environmental agreements. A key lesson learnt is that water governance needs to be realised through multiple policy tools and approaches. In particular, INEA articles provide evidence that alternative economic measures for valuing water are increasingly important. This point is best exemplified in the case of a long-standing water quality dispute in the Rhine. Cost-sharing was devised to share the burden of chloride pollution, when the polluter-pays-principle resulted in a deadlock of negotiations between France and the Netherlands (Dieperink, 2011). Extending the idea of valuing and quantifying benefits of water that eclipse polluter pay principles may hold promise for resolving transboundary watercourse problems. Macatangay and Rieu-Clarke (2018) analysed ecosystem services within a transboundary context and illustrated how this can highlight the value of benefits of cooperation over transboundary water. Studies show that analysis of incomplete or missing markets in relation to ecosystem service valuation and consideration of potential negative externalities continue to be important aspects of water agreements.

Early INEA articles recognised that water regarded as a purely private economic good sits uneasily with water as a guaranteed good accessed through human rights (Opschoor, 2006). However, categorisations of public, private, merit or economic good may actually be too limiting and fail to capture the full range of values associated with water (Ingram, 2006). Instead, water is intimately tied with community, culture and identity and can be a symbol for security, self-determination and preferred lifestyles. A later INEA article of Hurlbert (2020) recognised an alternative is to consider water as a commons, non-excludable but rivalrous, and to be shared. Furthermore, seeing water as a commons could enhance decentralised decision-making: local governments are encouraged to adopt democratic processes and procedures (Ingram, 2006); stakeholder are better engaged in decision-making (Hurlbert, 2020). These INEA insights have been subsequently supported by claims in wider literature about framing water as a commons that may benefit the issue of financing WASH, for example, where the situation is not so much who the financiers should be as the quality of institutional governance (Pories et al., 2019).

The economic dimension of providing WASH is well documented in the wider literature but insights from INEA suggest a need to strengthen supporting regulatory institutions. Documenting the lack of access to water globally, Tecco (2008) critiqued PPPs as a means for providing funding and extending water services. PPPs aim for enhanced efficiency but several factors need to be in place: tariff setting and balancing risks within PPP contracts and a well-functioning legal system. However, PPPs are not a substitute for strong effective local governance. In many countries, donor aid in addition to central government funding will be required (Tecco, 2008). This finding suggests that in order to make PPPs a success, efforts are required to establish conditions in which regulatory instruments can be enhanced. Subsequent scholarship confirms this point, including Hutton and Chase (2017) which documents the need to strengthen the enabling environment of PPPs through regulations, laws, resources and interventions.

### Insights on politics

Out of the articles reviewed, 19 articles place politics and political dimensions of governing water as its central analytical focus. These articles, along with others that deal more broadly on global governance of the environment, show that INEA has been a platform for the growth of scholarly debate on the contestations of governing water particularly in international contexts. An important contribution of the INEA water scholarship has been the examination of power and power asymmetries between transboundary basin states. Power asymmetry between states relates to both the material and discursive means that states exert in order to control water allocation. Such power asymmetry influences treaty design through varying tactics and strategies of hegemonic control. For example, treaty design might be curtailed as a result of sanctioning the discourse on treaty scope or focus. Power asymmetry can also manifest in differences of capacity to negotiate, to command funds, or to gain political support and alliances of other basin states such that treaties are designed to one’s favour (Zeitoun & Mirumachi, 2008). Most notably, Zeitoun and Mirumachi (2008) critiqued notions of ‘water conflict’ and ‘water cooperation’, unpacking the problematic normative assumptions that blindly encourage cooperation without due regard to the vested power relations that constrain equity.

A singular lesson from these pioneering studies is that power asymmetry is not fixed, and instead subject to change. The extensive three-part analysis on power asymmetry in hydro-hegemony not only examined the way power is consolidated to control water resources but also analysed the means of contesting and challenging such power (Zeitoun & Mirumachi, 2008; Zeitoun et al., 2011, 2017). In fact, contest and compliance coexist such that efforts to maintain the status quo of water allocation is always in tension with efforts to change it (Zeitoun et al., 2017). These findings indicate that power works to not only exclude actors from decision-making but also to challenge the status quo, thus creating a situation where control over water resources is not always guaranteed. Consequently, it can be said that simply seeking efficiency of policy instruments and principles may not be enough, because they do not sufficiently tackle the vested interests and power relations that shape these instruments in the first place. Because power drives mechanisms employed to contest (or comply), policies to enable actors cannot be blind to such differences of power.

Power is exercised particularly effectively when water is securitised by the state so as to influence policy decisions. Fischhendler (2015) clarified multiple mechanisms to securitise water that are set into motion by conditions of water scarcity and socio-political contexts, and in moments of crisis. Early work from the journal such as Fox and Sneddon (2007) highlighted that environmental agreements impose a certain socially constructed notion of state control over water. This works to securitise the environment such that state interests and sovereign integrity are preserved at the expense of ecological damage and livelihood impacts. Later, Urquijo et al. (2015) raised similar critiques of the state– in their case of the central Spanish authority securitising at times of drought—for setting up legal arrangements that would ultimately further economic development interests. In the case of Cyprus, securitising water has resulted in action to deal with water scarcity but only in a fragmented, unsustainable fashion. This means that collaborative approaches as well as taking into account biophysical and ecological considerations are limited at best (Zikos et al., 2015). Rendering water technical is another way of securitising water, as examined in examples from the Middle East by Weinthal et al. (2015). These analyses of state power have contributed to knowledge of agency in a context of hydro-hegemony (Conker & Hussein, 2020; Hussein & Grandi, 2017). The reason why international environmental agreements have yet to live up to their promise can be examined by the way environmental agreements ascribe an agency to the state control and power over water.

Lessons learnt in leveraging international environmental agreements suggest that agency needs to be reviewed in two ways. First, agency needs to be understood as exercised across multiple scales. Domestic pressure can constrain central governments in taking overtly hegemonic action (Petersen-Perlman & Fischhendler, 2018) or shift discourses (Zawahri & Hensengerth, 2012). Changes in political party at the domestic level or influences of external actors can lead to adjustments in foreign policy and regional relations, reshaping transboundary relations as well (Suhardiman & Giordano, 2012; Warner, 2012). Consequently, politics play out at domestic and international scales akin to ‘multiple chessboard games’ (Warner & Zawahri, 2012) and even at multiple levels, including regional scales (Jacobs, 2012). Second, the exercise of agency influences how existing rules, regulations and policy frameworks can be adapted. This has implications when policy coordination is needed across scales on interlinked issues of water, food and energy. Such processes will fundamentally require stakeholder participation in decision-making that is much more inclusive (Sharma & Kumar, 2020).

### Cross-disciplinary insights

INEA scholarship since its inception has advanced cross-disciplinary integration and insights. Our review of the 58 INEA water articles uncovered four main categories of cross-disciplinary insights. The first insight arises from connections between economics, law, and politics in relation to the framing of water as a good and constrain equity (Zeitoun & Mirumachi consequent legal and political implications. The second relates to policy instruments and their interactions contributing to ‘policy mixes’. The third relates to the nascent consideration of water ‘indicators’ and measurements across disciplines. Lastly, there are important cross-disciplinary insights on equity and water governance.

First, the framing of water (public, private etc.) discussed above extends cross-disciplinary insights pertaining to economic arrangements and legal agreements. Administrative law regimes and autonomous civil legal processes dominate in commodity frames, whereas international treaty law and human rights inform water and commons frames (although in some geographies and times both frames can co-exist) (Hurlbert, 2020). Commons are consistent with state water supply; commodity with private sector or PPPs. The commodity frame is reflected in de facto trade regimes (Conti & Gupta, 2016). However, this by no means implies consistent market frames follow commodity or market relations (Opschoor, 2006). Market-based approaches to water can be effected in a transboundary treaty regime fostering collective action within the law of international watercourses (Macatangay & Rieu-Clarke, 2018).

Framing water as a ‘political good’ beyond an economic and legal frame also has implications. Insights of economics and law are grounded in the politicised nature of the water sector, in which water as a ‘political good’ is contested (Opschoor, 2006; Schouten & Schwartz, 2006). This frame allows for practices that soften legal supply agreements—for example using rights as guideposts in service delivery and ensuring the private sector engage in transparent, accountable and participatory processes are key insights (Hurlbert, 2020). One of the most important insights arising from these multiple frames is the cautionary red flag that must be extolled in unpacking notions advancing frames such as commons, securitisation, and cooperation without unpacking vested power relations that constrain equity (Zeitoun & Mirumachi, 2008), a point which we shall return below. These cross-disciplinary insights illustrate that there is no one frame for water but multiple frames co-existing within varying economic and legal relations at plural scales.

Second, our review of INEA literature advances understanding of the use of policy instruments. Specifically, policy instruments are increasingly being used in conjunction with one another, and these instruments bridge legal, political and economic dimensions. International environmental agreements work alongside legal regulatory frameworks, most notably international water law. There has been advancement of the principle of no significant harm and underlying principle of a community of interest in water, as previously discussed. The INEA literature offers other examples of instrument combinations and mixes. Environmental impact assessments are increasingly taken up as legal instruments to settle disputes and resolve political tensions (Tignio & Bréthaut, 2020). Pursuing mediation and exploring economic ‘values’ underpinning a long-standing water quality dispute in the Rhine resulted in cost sharing between the two disputing nations (Dieperink, 2011). The lesson learnt from this case is that innovative ‘exchangeable’ interests can be created by thinking beyond or in addition to traditional legal instruments through dialogues around values and principles. Policy mixes prove to be effective as expanded economic tools can be complemented with legal ones such as arbitration, so that a limited issue of settlement of accounts (and not legality or liability for discharges) is possible. Combining economic analysis with legal instruments offers opportunities for the identification of incomplete or missing markets in relation to ecosystem service valuation, and consideration of potential negative externalities.

Another example is the supplement of legal instruments surrounding finance, planning, construction and operation of transboundary hydropower projects with guidance on safeguarding social and environmental concerns underscored by SDGs, WASH, and the no harm principle so that not only states but also private companies can follow through on their responsibilities (Rieu-Clarke, 2020). This policy instrument approach advances considerations of the synergies of water, energy and food, which need to be delivered as a ‘triplet’ in order to achieve SDGs (Pritee & Nithyanth, 2020). An important cross-disciplinary lesson learnt is that consideration of multiple and interacting policy instruments including treaties surrounding water, food, energy and biodiversity, international supply chains, prices and economics can illuminate issues of the water energy and food nexus. Policy mixes can support a more comprehensive approach to considering the entire water system, multiple users, and SDG obligations, which is important in tackling myopic thinking that is especially limiting in the policy sector.

Third, in the context of future climate change, there is a need for improved understanding of increasing impacts on vulnerable and marginalised people. Indicators of equity, minimum and maximum standards, and water distribution will be increasingly important. A small number of studies in INEA consider some of these water indicators, often relating to quantity or quality. Jaspers (2001) identified the need for indicators of minimum flows across state boundaries and integrated approaches to the study and management of water. Furthermore, engaging people in these processes is necessary to address water issues in Africa. Johns et al. (2018) measured the Great Lakes water regime effectiveness in relation to the objectives of the North American Great Lakes Water Quality agreement. These objectives provide indicators on allowing swimming and recreational use (unrestricted by environmental quality concerns) and on being free from aquatic invasive species. Conti and Gupta (2016) considered indicators of groundwater quality and quantity, significant harm to others, and measures to apply the precautionary principle (however they conclude specific measures as missing in the literature and data in water management practices). Jiménez-Madrid et al. (2018) proposed a system of indicators for operationalising conservation, safeguard measures and associated risk pressures/vulnerabilities in relation to groundwater that impacts GDP. As such, INEA has begun to build up studies on indicators to set minimum or maximum standards in relation to water quantity, water quality or other criteria, albeit more research evaluating and synthesising indicators is warranted.

Fourth, governance frameworks are an enduring and consistent concern in INEA scholarship. The INEA literature provides insights into tensions between politics, economics and law. For example, it is evident that there are in fact limits of privatisation and commoditisation of water as solutions for global water problems (contrary to almost two decades of many water professionals and the World Bank community arguing otherwise) (Schouten & Schwartz, 2006). This is because equity issues remain unresolved. The role of the private sector is questioned when poor people rely on informal infrastructure or in cases where increasing urban supply is at the expense of lifestyles and economies in pastoral areas (Ingram, 2006). It has been criticised that there is a lack of transparency and human rights are not used as guideposts for better governance (Gupta & Pahl-Wostl, 2013).

The studies reviewed focus on instruments and practices that encourage fairness, transparency and information exchange, as well as enhanced treaty design. International agreements attempt to mediate considerations of law, politics and economics, taking up tools and approaches that advance equity. The INEA studies on agreements to govern climate change and water are also instructive on the intersections of law, politics and economics. While the length and scale at which institutionalisation has occurred may be different between water and climate, Gupta and Lebel (2010) encourage multi-disciplinary governance frameworks that include political science, law, economics, sociology, geography and natural science.

Key INEA learnings concern stakeholder engagement in water governance and the value of social learning. Ingram (2006) points out that water ‘resources’ and ‘democracy’ have intimate association and more salience than efficiency and market relations of water management. In contexts of climate uncertainty, inclusivity of stakeholders has benefits for enhanced decision-making because knowledge can be shared by engaging individuals and organisations at multiple scales (Lebel, Grothmann, et al., 2010; Lebel, Xu, et al., 2010). In other words, for adaptative governance, it is not only the dialogue between people but also the knowledge exchange that can be valuable. Insights from INEA studies contribute to the wider scholarship on the science-policy interface, delving into the kinds of knowledge, data and information that can be useful. Citizen engagement can help address the challenges in international water law, which can be about its interpretation or implementation, as raised by Schmeier and Gupta (2020).

Social learning that encourages adaptiveness at different scales is beneficial for a number of reasons: to cope with informational uncertainty; to reduce normative uncertainty; to build consensus on criteria and indicators for monitoring and evaluation; to empower stakeholders to take adaptive actions; to reduce conflicts to identify synergies between adaptations; and to improve fairness of decisions and actions (Lebel, Grothmann, et al., 2010). However, there is a caveat on these benefits when there is a great diversity of interests. This is because environmental knowledge is crucial but often discounted because of opposing economic interests (Rosendal et al., 2019). The wider literature also raises caution about degree to which scientific knowledge improves adaptive capacity (Lemos, 2015).

Adapting to changes brings about a new set of politics, centring around uncertainty. There are new research questions to be posed on the implications of adaptation and how they impact equity, efficiency and effectiveness of water governance. These new research questions can provide policy insights on distributive justice and human security that result in improved access and allocation to water, energy and food. Considerations of international agreements will not make real change without consideration of power dynamics, disparity of equity of access and intersectionalities evidencing vulnerability (MacArthur et al., 2020). It is true that inclusion in decision-making processes does not always guarantee more equity (Zeitoun et al., 2011). However, attention to discursive practices and constructions, for example on WASH and indigenous and marginalised, can help in remedying inequity (Jiménez et al., 2014). Consequently, a vital lesson learnt from the INEA literature is the importance of beginning *any* analysis with an understanding of vested power that constrains equity. The large number of studies on transboundary waters published in INEA confirm that much learning can be missed without such considerations on the political context and overarching power relations.

## Conclusion

The key lessons learnt over the past twenty years of INEA scholarship focus on (i) innovation and continual challenges of ensuring water equity; (ii) use of multiple policy instruments that advance equitable sustainable development of water; and (iii) adaptive features of water governance in the context of climate change and uncertainty. These lessons suggest that rethinking is required for water governance. It is clear that treating equity as a ‘checklist’ or having a simple focus on government efficiency in implementing international environmental agreements make participatory governance effectively meaningless. The implementation of policy mixes needs to be set against a backdrop where there is essential understanding of how power asymmetry in treaties and international agreements manifest, lest cooperation parade as equitable but reify vested power relations that constrain inequity.

Importantly, alternative discourses that engage multiple actors, scales and domains are urgently needed to address historic siloed thinking and new research strategies. The findings from INEA studies provide cautionary considerations in relation to power dynamics of actors and their agency, the politics of contested water relations, and vested interests in securitising water to advance state control and reduce collaborative approaches that might enhance ecological considerations and local livelihoods. Policy debates, including water diplomacy efforts, need to consider alternatives to such dominant practices of water governance (Zeitoun et al., 2020). Focus needs to be on how alternatives can emerge through the engagement and empowerment of stakeholders. There is also a need to ask how water can be valued differently and leverage legal and economic policy instruments. Doing so would shed light not only on the technical management of water resources and its associated resources such as land, but also on socio-economic drivers in and outside the water sector, global frameworks including climate and the interface of domestic-international politics on environmental matters. Climate change, limits on adaptation, human migration, trade relations, supply chain failures, extreme events (drought, flood, fire) all have important water dimensions requiring focused attention. New issues of concern for international agreements are also emerging. For example, the COVID-19 pandemic has highlighted lack of WASH in connection with health. In addition, the international attention to ocean plastic refuse offers new opportunities for environmental remediation and reduction of the fossil fuel industry generating it. These emerging discussions offer new opportunities for research directions in INEA. However, research should not lose focus on the transformation of existing international water agreements and the role of states. Nation states still have an important role to play in advancing equitable water allocations and access, especially in relation to recognising WASH in nation state legal frameworks. With these future research insights, there may be a step change in the way society engages with the environment.

## Abbreviations

INEA: International Environmental Agreements: Politics, Law and Economics
IWRM: Integrated water resources management
PPP: public private partnership
SDG: Sustainable Development Goal
WASH: Water, sanitation and hygiene
